# An innovative sealed shoe to off-load and heal diabetic forefoot ulcers – a feasibility study

**DOI:** 10.1080/2000625X.2017.1348178

**Published:** 2017-07-25

**Authors:** Gustav Jarl, Roy Tranberg

**Affiliations:** ^a^ Department of Prosthetics and Orthotics, Faculty of Medicine and Health, Örebro University, Örebro, Sweden; ^b^ University Health Care Research Center, Faculty of Medicine and Health, Örebro University, Örebro, Sweden; ^c^ Department of Orthopaedics, Institute of Clinical Sciences, The Sahlgrenska Academy at University of Gothenburg, Gothenburg, Sweden

**Keywords:** Diabetic foot, ulcer, shoes, orthotic devices, patient compliance, diabetes mellitus, diabetes complications

## Abstract

**Background**: Non-removable knee-high devices are the gold standard to treat diabetic foot ulcers located on the plantar forefoot, but they immobilize the ankle, which restricts daily life activities and has negative effects on joint functioning.

**Objective**: To investigate the feasibility of sealing a therapeutic shoe to off-load and heal diabetic forefoot ulcers.

**Design**: A case series of seven men with type 2 diabetes and a metatarsal head ulcer were prescribed therapeutic shoes and custom-made insoles. The shoe was sealed with a plastic band. Off-loading was assessed with the F-scan pressure measurement system. Adherence to wearing the shoe was assessed with a temperature sensor and by documenting the status of the seal.

**Results**: The off-loading was effective and all ulcers healed. Median time to healing was 56 days (range 8–160). Complications were secondary ulcer (*n* = 1) and plantar hematoma (*n* = 1). Five of seven participants did not disturb the seal.

**Conclusions**: Sealing a therapeutic shoe is a feasible way to off-load and heal forefoot ulcers. A controlled trial is needed to compare the effectiveness and safety of a sealed shoe to other non-removable devices.

## Background

Approximately 20% of diabetic foot ulcers are located on the plantar forefoot [1,2]. Total contact casts (TCCs) and cast walkers rendered irremovable are the gold standard to off-load and heal these ulcers [3–5], but they immobilize the ankle, which restricts daily life activities [3] and has negative effects on joint functioning [6]. A possible alternative would be an ankle-high device rendered irremovable, and a case study reported successful result with using a plaster shoe instead of a knee-high TCC [7]. To the authors’ knowledge, this idea has not been extended to therapeutic shoes. The study aim was to investigate the feasibility of using a therapeutic shoe, rendered irremovable, to off-load and heal forefoot ulcers.

## Design

The study was a case series approved by the Regional Ethics Committee Review Board of Uppsala. Inclusion criteria were diabetes mellitus and a metatarsal head (MTH) ulcer. Exclusion criteria were toe pressure <40 mmHg, active Charcot foot, and deformity that prevented use of off-the-shelf therapeutic shoes.

Presence of neuropathy was investigated with a 10 g monofilament under the first toe and the first and fifth MTH, and with a tuning fork (128 Hz) on the dorsal side of the first toe, following the guidelines of the International Working Group on the Diabetic Foot [8]. Passive dorsal flexion range of motion was investigated in the ankle (with knee extended) and first toe on the ulcerated foot. Participants had their toe pressure measured and received extra-depth therapeutic roller shoes. One author (GJ) made casts of the participants’ feet and produced custom-made insoles of 14 mm ethylene–vinyl acetate (two layers with a hardness of 50 and 20 Shore) with 3 mm microcellular urethane glued on the top surface. A sensor (Orthotimer, Balingen, Germany) measuring the temperature every 15 min was placed under the urethane layer. The insoles were ground to the shoes leaving 10 mm of material under the heel and 15 mm under the MTHs. The ulcer was off-loaded by grinding the insole from the underside on the location of the ulcer until the urethane layer was exposed. An F-scan system [9] was used to measure plantar pressures as the person walked three times, 8 s per time, at a self-selected speed on a level surface. The insoles were then adjusted as necessary and the pressure measurement repeated to ensure that the ulcer was off-loaded and that there were no excessive pressures.

**Figure 1.**
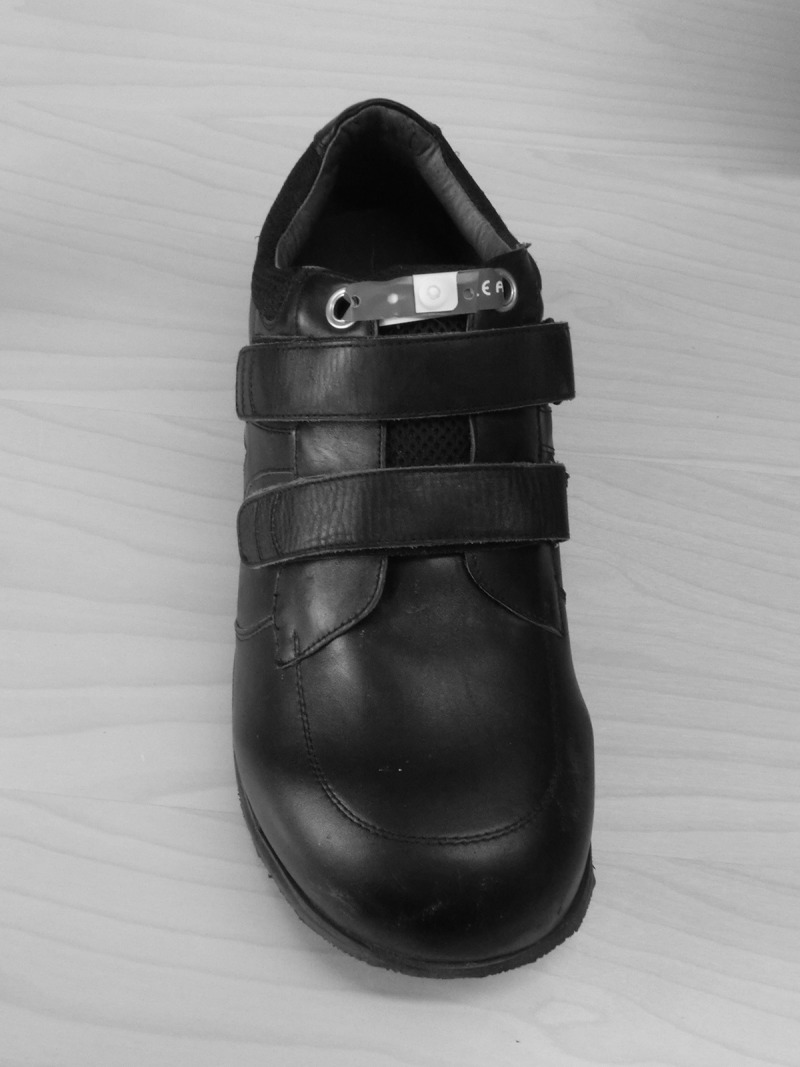
The therapeutic shoe was sealed by threading a plastic band (Brace-lok, DJO Nordic, Malmö, Sweden) through two holes; one on each side of the shoe’s opening.

Participants broke in the shoes by wearing them for 7 days and one night. After this, the shoe on the ulcerated foot was sealed with a plastic band (Figure 1). Participants were instructed to break the seal and remove the shoe only if they experienced any sign of deterioration of the foot or general condition, or if something had fallen into the shoe. Thus, participants wore the shoe day and night and were free to walk as much as they desired during the treatment period. Shoe covers were used when sleeping and showering. A primary care nurse re-dressed the ulcer (typically 1–2 times/week) and documented whether the seal was broken or not, as well as complications and complaints before sealing the shoe again. Every 14 days, a podiatrist revised the ulcer using sharp debridement. Healing was defined as the date when the ulcer was completely epithelized if it was still epithelized 14 days later [10]. After healing, participants filled in a questionnaire about advantages and disadvantages with a sealed shoe compared with removable shoes and TCCs.

## Results

Seven men with type 2 diabetes for >10 years participated (Table 1). No participant could feel the monofilament or tuning fork. The ulcers had Wagner grade 1 and 2. Median ulcer duration was 1.0 years and median ulcer size (longest diameter multiplied by longest perpendicular diameter) was 0.5 cm^2^.

**Table 1.** Characteristics of participants and ulcers.

Sex	Age, years	Ankle dorsal flexion ROM*, degrees	First toe dorsal flexion ROM*, degrees	Toe pressure*, mmHg	Ulcer location	Wagner grade	Ulcer size**, cm^2^	Ulcer duration, years	Time to healing, days
Men (*n* = 7)	63	5 (−10–10)	40 (30–70)	112	MTH 1 (*n* = 4)	Grade 1 (*n* = 4)	0.5	1.0	56
Women (*n* = 0)	(35–80)			(55–165)	MTH 3 (*n* = 1)	Grade 2 (*n* = 3)	(0.2–2.0)	(0.3–4.7)	(8–160)
					MTH 4 (*n* = 1)				
					MTH 5 (n = 1)				

Continuous variables are medians (min–max). *On the ulcerated foot. **Calculated as longest diameter multiplied by longest perpendicular diameter. MTH: metatarsal head. ROM: range of motion

All ulcers healed. Time from sealing of the shoe to healing was 8–66 days for six ulcers and 160 days for one ulcer, median 56 days. The most frequent complications were considered as minor, such as redness of the skin. One participant did, however, develop a secondary ulcer on the dorsum of the foot. After this ulcer had healed, he rejoined the study, now with a seal that allowed adjustment of the Velcro to accommodate the varying edema. Another participant developed a plantar hematoma, but it healed without ulceration.

The median peak pressure on the ulcer was 116 kPa (62–192 kPa) when walking with the shoe. Five participants respected the seal and only removed the shoe on legitimate occasions such as medical examinations. One participant broke the seal and removed the shoe at night, but was very adherent in the daytime. Another participant removed the shoe on a few occasions without breaking the seal. The temperature data were useful for assessing adherence, but in some cases equivocal: a temperature drop could reflect either that the participant removed the shoe or went outdoors in cold weather. All participants had previously used removable therapeutic shoes, and reported advantages with a sealed shoe were reasonably quick healing (*n* = 1) and not being tempted to walk without the shoe (*n* = 1). Disadvantages were that the shoe was hot/sweaty (*n* = 1) and caused difficulties in dressing (*n* = 5), showering (*n* = 1) and sleeping (*n* = 1). Four participants had previously used a TCC, and reported that advantages with a sealed shoe were being able to work and move around almost normally without crutches (*n* = 1) and not having to change casts (*n* = 1). No disadvantages were reported.

## Discussion

This study supports the feasibility of sealing a therapeutic shoe to off-load and heal diabetic forefoot ulcers.

Guidelines [3,11,12] recommend non-removable knee-high devices to heal forefoot ulcers because of their off-loading capacity and ‘forced adherence’. Our pressure measurements demonstrated that a therapeutic shoe with an optimized insole can effectively off-load at least small forefoot ulcers. However, one plantar hematoma developed, which indicated that shoes might not be feasible for off-loading all ulcers. Although sealing did not result in 100% adherence, adherence reached levels that allowed most ulcers to heal within a reasonably short time. Still, we cannot exclude that adherence partly improved because participants knew that the temperature sensor would register any removal of the shoe.

Guidelines [3,11] recommend therapeutic shoes to prevent reulceration after healing. However, if reulceration occurs after changing from a non-removable knee-high device to shoes, the person might attribute the new ulcer to the shoes per se rather than to low adherence to wearing them. In contrast, if reulceration occurs after removing a seal from the shoe this can serve to educate the patient of the importance of adherence. Hopefully, this can empower patients to break the vicious cycle of reulceration, effective treatment, and non-effective prevention.

One participant developed a secondary ulcer and another removed the shoe at nighttime, in both cases because edema had caused pressure on the foot. Thus, special caution should be taken if edema is present. Notably, the participant who developed the ulcer had previously developed an ulcer in the same location when wearing a TCC.

This study has some limitations. First, no strict criterion was used to decide whether the ulcers were adequately off-loaded or not. Second, the sample was small and included men with type 2 diabetes only. Acknowledging these limitations, sealed shoes are an interesting avenue for future research as they include some of the advantages of non-removable knee-high devices and avoid some of the disadvantages that limit their clinical use [13–15]. For example, a sealed shoe does not immobilize the ankle joint. In addition, a sealed shoe might be a more attractive alternative in low- and middle-income countries as no special technical expertise is needed for application of a TCC. Furthermore, the same device can be used to both prevent and heal ulcers, thus limiting costs. To investigate the effectiveness and safety of this concept with other non-removable devices, a larger randomized controlled study is needed.

## Geolocation information

The study was conducted at Örebro University Hospital, Örebro, Sweden.
